# Quantitative and Predictive Genetic Parts for Plant Synthetic Biology

**DOI:** 10.3389/fpls.2020.512526

**Published:** 2020-10-06

**Authors:** Diane M. McCarthy, June I. Medford

**Affiliations:** Department of Biology, Colorado State University, Fort Collins, CO, United States

**Keywords:** synthetic biology, transfer function, orthogonal, genetic circuit, mathematical modeling, plant synthetic biology

## Abstract

Plant synthetic biology aims to harness the natural abilities of plants and to turn them to new purposes. A primary goal of plant synthetic biology is to produce predictable and programmable genetic circuits from simple regulatory elements and well-characterized genetic components. The number of available DNA parts for plants is increasing, and the methods for rapid quantitative characterization are being developed, but the field of plant synthetic biology is still in its early stages. We here describe methods used to describe the quantitative properties of genetic components needed for plant synthetic biology. Once the quantitative properties and transfer function of a variety of genetic parts are known, computers can select the optimal components to assemble into functional devices, such as toggle switches and positive feedback circuits. However, while the variety of circuits and traits that can be put into plants are limitless, doing synthetic biology in plants poses unique challenges. Plants are composed of differentiated cells and tissues, each representing potentially unique regulatory or developmental contexts to introduced synthetic genetic circuits. Further, plants have evolved to be highly sensitive to environmental influences, such as light or temperature, any of which can affect the quantitative function of individual parts or whole circuits. Measuring the function of plant components within the context of a plant cell and, ideally, in a living plant, will be essential to using these components in gene circuits with predictable function. Mathematical modeling will be needed to account for the variety of contexts a genetic part will experience in different plant tissues or environments. With such understanding in hand, it may be possible to redesign plant traits to serve human and environmental needs.

## Introduction

Plants are an attractive platform for engineered traits because of their advanced ability to adapt to their environments, variety of growth forms, and natural ability to produce useful secondary compounds. They also allow us to avoid the ethical issues often associated with working with mammalian systems. Plants naturally use complex types of computation to process and respond to environmental inputs using genetic circuits. For example, they use information such as day length and developmental age to “compute” whether to flower. A goal of plant synthetic biology is to harness these natural abilities of plants and turn them into new purposes. The natural computation abilities of plants have been likened to those used in electronics. Historically, understanding the key principles behind electronic computation took us from room-sized vacuum-tube computers to small, powerful cell phones in only a few decades. These principles include digital logic, quantitative analysis, and a vast library of modular DNA parts or components with well-characterized function. Types of parts can include promoters, 5' and 3' UTRs, coding sequences, terminators, activators, and repressors. Plant synthetic biology similarly aims to produce predictable and programmable genetic circuits from combinations of simple regulatory elements and well-characterized genetic parts. While we are developing parts libraries and methods for rapid quantitative characterization, the field of plant synthetic biology is still in its early stages. We here describe methods used to describe the quantitative properties of genetic parts needed for plant synthetic biology. Once the quantitative properties and transfer function of a variety of genetic parts are known, computers can select the optimal parts to assemble into functional devices, such as toggle switches and positive feedback circuits. With such understanding in hand, it may be possible to redesign plant traits to serve human and environmental needs.

## Considerations for Selecting Parts

The biological concept of predictable and programmable genetic function can be traced to Jacob and Monod’s Nobel prize-winning work on the *lac* operon in bacteria (Jacob and Monod, 1961a,b). They proposed that gene circuits with “virtually any desired property” could be constructed from the simple regulatory elements found in genes. Understanding the *lac* operon was key to developing a quantitative predictive understanding of gene regulation (reviewed in Santillan and Mackey, 2008; Garcia et al., 2010) and laid the groundwork for future work in the engineering of synthetic regulatory networks with predictable function. Another 40 years passed before the first synthetic gene circuits with predictable function were produced using simple bacterial plasmids (Elowitz and Leibler, 2000; Gardner et al., 2000). More time would pass before synthetic genetic circuits with predictable function could be implemented in eukaryotic cells (Kramer et al., 2004), and it would be even longer before predictable circuits were possible in multicellular organisms, such as plants (Schaumberg et al., 2015).

### Parts Libraries for Plants

Genetic parts can be mined from prokaryotes, eukaryotes, and existing libraries of their parts. We can also construct new synthetic parts by combining genetic elements from myriad source organisms. In the past, simple traits like herbicide and insect resistance were engineered into plants (Mazur and Falco, 1989; O’Callaghan et al., 2004) with quantitatively predictable function, but in general, it was difficult to identify plant traits whose functions would be predictable in other host species.

Synthetic biology circuit construction benefits from the availability of genetic parts of different function and strength that can be assembled in well-defined ways to accomplish outcomes. This methodology parallels that seen in mechanical and electrical engineering, where development of a new device or circuit begins with a library of existing parts of known function. These genetic parts may originate from prokaryotes or eukaryotes, or be synthetically designed, and can have varying strengths. Biochemical parameters that can be measured for each part include binding affinities, transcriptional rate constants, quantity of repressor or activator, promoter strength, rate of protein synthesis, and RNA or protein degradation rates. Many gene parts have been produced and characterized for *Escherichia coli* and yeast (Fischbach and Voigt, 2010; Lou et al., 2012; Moon et al., 2012; Cambray et al., 2013; Chen et al., 2013b; Kosuri et al., 2013; Mutalik et al., 2013a,b; Casini et al., 2014; Michener and Smolke, 2014; Lee et al., 2015). Such well-characterized parts have already been used to produce circuits with predictable function (Moon et al., 2012; Bonnet et al., 2013; Cambray et al., 2013; Chen et al., 2013b; Kosuri et al., 2013; Mutalik et al., 2013a,b). Characterization of eukaryotic parts lags that of prokaryotes. For prokaryotes, outstanding work has been accomplished (Clancy and Voigt, 2010; Fischbach and Voigt, 2010; Nielsen et al., 2016). In mammalian systems, parts have also been developed including using CRISPR technologies (Qi et al., 2012; Slusarczyk et al., 2012).

Design of novel synthetic parts offers a rich and highly customizable source of circuit components. Libraries containing characterized cis elements such as DNA binding domains and effector domains can be mined to construct synthetic transcription factors and other parts that are new to nature. Kassaw et al. (2018) reviewed genetic element categories and design principles underlying assembly of a variety of synthetic parts in plants, including transcription factors and repressible promoters. With access to a variety of elements of varying strength and binding specificity coupled with rules for assembly to achieve different functions, the potential number of parts available for plant circuits is virtually unlimited.

### Computational Protein Design

A powerful source of parts for plant synthetic biology is computational protein design. The ability to rationally design novel proteins to fit specific purposes has made substantial advances. We have already seen computationally designed customizable enzymes and ligand-dependent transcriptional activators, including in plants (Koga et al., 2012; Kiss et al., 2013; Tinberg et al., 2013; Feng et al., 2015). Until recently, existing proteins with characteristics approximated to those of the desired protein were modified to bind the intended target. The technology has now been developed to enable *de novo* protein design, thus eliminating the constraints represented by repurposing existing proteins (Huang et al., 2016). Theoretically, a protein to perform any function should be possible. In the future, the ability to produce new parts (promoters, effectors, coding sequences, etc.) *de novo* based on fundamental design principles could lead to assembly of highly customizable synthetic circuits in plants.

### Orthogonality

Another consideration when selecting genetic parts is orthogonality. Orthogonality, the ability of genetic parts and circuits to function independently of each other and of the regulatory functions of the host, is key to synthetic biology. Orthogonal parts can be borrowed whole or in part from systems other than the intended host species, most often bacterial, yeast, or plant viral sequences. Plant bacterial and viral pathogens have provided many useful plant parts (Saunders and Lomonossoff, 2013; Bruckner et al., 2015). Regulatory elements that provide sensitivity to factors routinely experienced by plants, including light, drought, and temperature, can be obtained from algal, fungal, or photosynthetic microbes, thereby producing plant-like responses in an orthogonal way. Orthogonality also increases the specificity of a part’s function (Lucks et al., 2008; Khalil et al., 2012; Morey et al., 2012; Chen et al., 2013b). We can also engineer synthetic orthogonal parts by customizing DNA binding elements to specific promoter elements (TAL effectors and zinc fingers). The dCas9 enzyme has been used to engineer transcriptional activators and repressors (Lowder et al., 2015; Piatek et al., 2015).

Plants themselves can also be mined for regulatory elements (Dey et al., 2015) or genetic parts, though they are less likely to be naturally orthogonal to their intended host plant than, for instance, a yeast element. To provide orthogonality to a plant element used in a plant synthetic circuit, one typically must refactor the part. Refactoring is a process in which the genetic part’s native design parameters are simplified, and inefficiencies and endogenous regulation are removed, while still retaining the essential function of the part (Temme et al., 2012). The refactoring process can and should be done on genetic parts sourced from any species, and it can transform even a plant-derived sequence into an orthogonal sequence. Engineered synthetic parts and the sequences for *de novo* protein designs will likely be orthogonal to the host cell’s function without additional refactoring. Note that complete orthogonality is undesirable, as the synthetic circuit must interact with native cell machinery and regulators such as RNA polymerase. In addition, typical plant transformation techniques require use of a prokaryotic intermediary, such as *Agrobacterium*. Refactored plant parts and assembled circuits must remain compatible with the plasmids for *Agrobacterium*-mediated transformation or other plasmids used in transient assays (Saxena et al., 2016; Pasin et al., 2017).

### Quantitative Characterization

Whole plants take a long time to stably transform, so transient expression in protoplasts (plant cells) serves as a proxy (Ono et al., 2004; Sainsbury and Lomonossoff, 2014; Schaumberg et al., 2015). Standardized testing platforms provide accurate quantitative characterization of individual parts. Such data can then be deposited into parts libraries for broader use. Once a circuit has been designed, data about the functionality of each part become inputs into a model that predicts complete circuit function. Schaumberg et al. (2015) quantitatively characterized more than 220 synthetic repressors and repressible promoters for use in plant circuits. These parts were derived from yeast Gal4 (Giniger et al., 1985) and bacterial LexA (Schnarr et al., 1991) DNA binding domains fused with transcriptional repression domains from *Arabidopsis* (Ohta et al., 2001; Wang et al., 2007, 2011; Ikeda and Ohme-Takagi, 2009). The parts were tested and characterized in *Arabidopsis* and *Sorghum* protoplasts using a dual luciferase assay to quantify repressor production and repressible promoter function. Their methodology also incorporated a mathematical model that determined the input-output, or transfer, function characteristics of promoter-repressor pairs, and was capable of accounting for noise from plant systems. The methodology was validated in stably transformed *Arabidopsis* and *Sorghum* plants (Schaumberg et al., 2015). This general approach can be used to quantitatively characterize genetic parts for use in plants. However, while rapid testing of large numbers of plant parts is most readily accomplished in protoplasts, their function when stably transformed into whole plants may be different. Plants exhibit slow growth and long life cycles relative to bacteria, and have distinct cell and tissue types and developmental stages. To measure function in different cell types, we can derive protoplasts from different plant tissues, such as leaves or roots. However, these measurements will not tell us about function in whole tissues made of interconnected cell types, in plants facing heterogeneous environmental stimuli inside or outside a lab setting, or in untested cell types. These factors make quantitative characterization of their genetic parts, assembly into circuits, and testing those circuits challenging. Follow-up testing of parts and genetic circuit function in stably transformed whole plants is critical, though time-consuming.

In bacteria and cultured mammalian cells, quantitative data on genetic parts can be obtained using fluorescence-activated cell sorting (FACS) and fluorescence proteins (Fischbach and Voigt, 2010; Chen et al., 2013b; Stanton et al., 2013, 2014). A special consideration with using fluorescent proteins in plant cells such as protoplasts is that chlorophyll, a highly abundant fluorescent protein in plants, can overwhelm the desired output signal. Fluorescence proteins can be used for FACS in plant protoplasts but they must be carefully selected. Use of root protoplasts can help circumvent the chlorophyll problem (Evrard et al., 2012). Still, transient assays developed to date do not address plant tissue or organ specific gene expression. The parts characterization process needs to take into consideration the context within the organism, including the tissue or organ in which it is expressed, and the developmental stage. Transient assays in single cells are not capable of providing this level of detail.

### Transfer Functions

Transfer functions, or input-output functions, describe a plot or function of the ratio between input and output across a range of inputs. As applied to genetic parts, they plot the change in quantity of an output (e.g., a protein) over a range of input quantities (e.g., an activator). The curve can describe a change in gene expression, but can also describe other biological features, such as stability, abundance, or activity of a genetic part (Medford and Prasad, 2016). The shape of a transfer function is generally sigmoidal, indicating a rapid change between the output OFF state and ON state once some threshold level of input has been reached. The OFF and ON states are stable and exist over a broad range of input parameters, providing stability against a switch to the alternate state arising from noise on the input side. This combination of bistability and rapid state change produce a digital-like response. To measure the transfer function for a given gene part, the part is isolated in a testing platform, such as the dual-luciferase transient system described by Schaumberg et al. (2015). In that system, the “input” is a protein activator. When the gene part is activated, it begins transcription, leading to production of some quantity of output protein. In the case of screening individual parts, this output could be a reporter protein, such as luciferase or GFP. Any change in the part being tested, or in the elements comprising a circuit, might change the shape of the transfer function. Accurate characterization of parts *via* their transfer function is critical to the design of functional, predictable circuits (Medford and Prasad, 2016). Transfer functions also serve as inputs to computational simulations of assembled circuits in order to predict the circuits’ function, robustness, and properties. For example, the output of one part becomes the input for the next, so the dynamic ranges of the two parts must overlap in order for the second part to be activated. These simulations form the basis for mathematical modeling of circuit function and contribute to the iterative “design-build-test” workflow that is at the heart of synthetic biology. Parts identified in simulation as not optimal can be replaced by more suitable parts from the library or can be modified *via* additional *in silico* (e.g., refactoring and replacement of cis elements) or *in vitro* (e.g., directed evolution) processes.

### Circuit Assembly

Cells naturally encode regulatory multilayered regulatory genetic circuits that determine gene expression, and process and act on information. Once a library of quantitatively characterized genetic parts is available, genetic circuits can be constructed using computer programs that select combinations of parts to produce a circuit design with predictable function. Such circuits in plants include toggle switches and positive feedback circuits (Tamsir et al., 2011; Moon et al., 2012; Medford and Prasad, 2016). Predictable circuits can then be combined or layered to form more complex functions that can function in more than one species, as shown in Schaumberg et al. (2015). There are a variety of assembly languages available in synthetic biology (Beal et al., 2011, 2012) that can be used to define the parts and the “grammar” for assembling parts into meaningful, functional circuits. However, these languages have not been fully adopted by the plant community.

As the number of available characterized parts grows, and platforms for testing become more sensitive and accurate, the design principles for constructing parts and circuits will also improve. Multicellular eukaryotes are complex, and their genetic regulatory networks are not fully understood. Better parts plus better design principles will result in circuits that behave with greater predictable function and reliability in plants. Further, more precise parts-level function data will enable the use of biological computer-aided design (Bio-CAD) software tools that can fully automate parts selection and circuit construction (Khalil et al., 2012; Chen et al., 2013b; Nielsen et al., 2016). Software tools that have been used for construction of gene circuits in plants include GeneDesigner (Villalobos et al., 2006), CellModeller (Dupuy et al., 2007), BiopartsBuilder (Yang et al., 2015), GenoCAD (Coll et al., 2015), and GoldenBraid (Vazquez-Vilar et al., 2017). These and other tools provide a common syntax that enables modular construction of multiple circuit configurations out of available genetic parts. Patron et al. (2015) describe a Type IIS genetic syntax that employs the principles of parts reusability and standardization to enable circuit assembly.

After the circuits are designed, assembly of DNA fragments can be performed in a variety of ways (Gibson et al., 2008, 2009; Sarrion-Perdigones et al., 2013; Casini et al., 2014). Older cloning approaches left scars (short fragments of DNA) between DNA fragments that could interfere with quantitative function. Newer assembly methods such as Gibson assembly (Gibson et al., 2008, 2009) and AssemblX (Hochrein et al., 2017; Lukan et al., 2018) create scarless constructs. Methods to integrate and streamline the entire synthetic circuit construction process from parts selection to assembly have advanced, and we are close to having available “genetic compilers,” integrated systems that allow computerized selection of optimal parts to achieve a given function, along with robotics to select and assemble those parts (Clancy and Voigt, 2010; Kahl and Endy, 2013; Smanski et al., 2014). With the aid of these technologies, design and production of circuits will be faster and more accurate.

There are additional challenges at the circuit assembly stage. The presence of multiple copies of the same part in multilayered circuits can lead to homologous recombination (Chen et al., 2013b; Brophy and Voigt, 2014), reinforcing the need for available interchangeable parts that perform similar functions. A part’s context within the assembled circuit also affects its function. The behavior of individual parts is sensitive to their spatial arrangement and to the orientation of independent transcriptional units within a circuit. Parts obtained from multiple sources (e.g., bacterial or fungal) might behave differently together than when characterized in isolation. They will behave differently when tested in transient assays such as in protoplasts versus when stably transformed into plants and will likely have different function depending on plant species, tissue, or developmental stage. One way to counteract these sources of uncertainty is through the development of design principles such as genetic circuit isolation. Transcription blocks and insulators are known to prevent unintentional activation or silencing of transgenes in plants (reviewed in Singer et al., 2012). Some insulators function to block the interaction between a remote enhancer and a promoter. Other insulators are thought to force RNA polymerase off the DNA molecule. RNA processing tools such as ribozymes and CRISPR target sequences can act as insulators against context effects when inserted between the 5' UTR and the ribosome binding site, as has been shown in both prokaryotes and eukaryotes (Bashor and Collins, 2012; Lou et al., 2012; Qi et al., 2012).

## Plant Synthetic Biology Applications

By applying the framework developed by Schaumberg et al. (2015), it should be possible to rapidly characterize large numbers of parts and produce synthetic circuits or devices such as toggle switches and positive feedback circuits in plants. With these basic devices in hand, we can then assemble more complex and layered genetic circuits to achieve functions that are entirely new to nature and serve human needs.

### Toggle Switches

**Figure 1:**
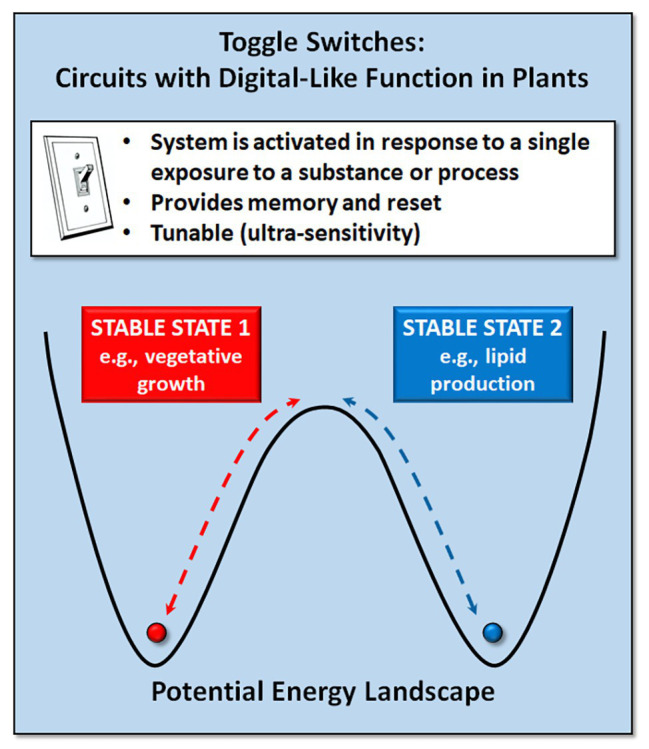
Genetic toggle switches provide the ability to reversibly switch between two stable states (e.g., vegetative growth vs. lipid production for biofuels).

A toggle switch or on-off switch is the simplest form of electrical circuit. A genetic toggle switch is a circuit that can produce two clearly different output states with a reversible transition between them (Figure 1). Such bistable switches, comparable to the binary states in electronic circuits, are important in decision making in natural genetic circuits. In higher eukaryotes, bistable switches underlie cellular decision processes, such as cell proliferation and differentiation and tissue patterning (Yao et al., 2008; Cruz-Ramírez et al., 2012; Gaillochet et al., 2015). In *Arabidopsis* roots, a bistable switch mediates expression of the SHORT ROOT (SHR) and SCARECROW (SCR) genes to ensure accurate spatiotemporal control of asymmetric cell division (Cruz-Ramírez et al., 2012). Similarly, the WUSCHEL (WUS) and CLAVATA (CLV) genes in plants act as a simple toggle switch, forming a bistable system that maintains shoot meristem homeostasis while also initiating organ primordia (Schoof et al., 2000; Zuo et al., 2002; Gallois et al., 2004).

**Figure 2:**
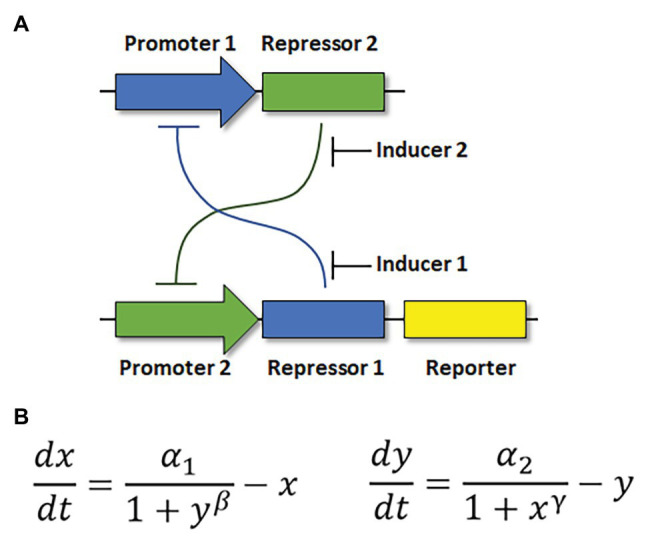
Structure of a genetic toggle switch in *Escherichia coli*. **(A)** The toggle circuit design consists of two constitutively active, mutually repressible promoters controlling expression of two repressors. **(B)** Mathematical equations describing the interactions of the genetic parts in **(A)**. *x*: concentration of repressor 1. *y*: concentration of repressor 2. *α*_1,2_: rate of synthesis of repressors 1 and 2. *β*, *γ*: cooperativity of repression from promoters 1 and 2, respectively. Adapted from Gardner et al. (2000).

Properly constructed, a bistable genetic switch permits sophisticated, computer-like function in a biological system (Fussenegger et al., 2000; Lebar et al., 2014). One of the first synthetic gene regulatory networks was a toggle switch in *E. coli* (Gardner et al., 2000). This switch was constructed from constitutively active, mutually repressible promoters (Figure 2A). Its construction was based on mathematical modeling that described how the promoters and repressors should be quantitatively balanced, and that the relationships between the parts should be non-linear in order to produce two stable states (Figure 2B). Other synthetic toggle switches have since been constructed in bacterial or mammalian cells (Atkinson et al., 2003; Kramer et al., 2004).

Gathering quantitative data for the transfer function is an important first step for assembly into devices such as toggle switches. Toggle switches in plants could be used in a variety of applications. For example, synthetic toggle switches can help regulate on-demand production of bioenergy traits such as seed oil deposition or increased biomass, or detection of pathogens or heavy metals. A number of bistable switches have been produced in prokaryotes and in mammalian cell culture (Gardner et al., 2000; Schoof et al., 2000; Kramer et al., 2004; Deans et al., 2007; Tan et al., 2009; Chen and Arkin, 2012). A mutual repressor-based toggle switch comprised of transcription-activator-like effector (TALE) DNA binding domains has been reported in mammalian cells (Lebar et al., 2014), but was found not to display bistability. Addition of a positive feedback loop did result in epigenetic bistability (Lebar et al., 2014). In an example in plant cells, a toggle-like switch based on a phytochrome-based red light responsive system was reported to be functional in *Nicotiana* protoplasts (Müller et al., 2014). However, this system is not predicted to be orthogonal in stably transformed plants. A synthetic toggle switch in a stably transformed *Arabidopsis* plants is in the works and should be described shortly. The circuit demonstrates the feasibility of designing and producing a bistable system in plants out of well-characterized parts, based on mathematical modeling to balance their expression.

### Positive Feedback Circuits

In nature, positive feedback systems are typically used to regulate sensitivity to an input, while negative feedback systems are typically used in nature to mediate responses or to reduce the effects of perturbations, thereby leading to stable states (Lestas et al., 2010; Chen et al., 2013a). Most synthetic feedback systems have been positive feedback systems. Natural feedback circuits can lead to bistable regulation of states such as undifferentiated and differentiated shoot apical meristem cells (Gaillochet et al., 2015), although unlike toggle switches, states induced by a positive feedback circuit are usually irreversible. These capabilities can be useful when designing new synthetic circuits. Feedback circuits are auto-regulatory in that a transcription factor controls its own expression, positively or negatively, at the transcriptional level. This step in the genetic circuit is the feedback loop. Its inclusion can dramatically reduce the time required for the circuit to reach steady state (Bansal et al., 2010). Feedback circuits, with their self-propagating feedback steps, are difficult to correctly balance. Leaky expression is a problem in positive feedback circuits, and careful balancing of genetic parts expression, as guided by the mathematical model, is required to produce bistability. The optimal combination of genetic parts to achieve bistability is more difficult to achieve in feedback circuits than in toggle switches (Chang et al., 2010). The availability of well-characterized genetic parts and mathematical modeling to assemble those parts into a circuit design is key.

An example of a useful positive feedback mechanism is increasing sensitivity, in other words enabling a cell to sense a small number of molecules and produce a response (Afroz and Beisel, 2013). This result is useful for applications such as sensing of external signals, where a small or transient exposure to a compound leads to amplification of a response that is proportionally large relative to the initial signal. This was done in *Arabidopsis* and lettuce protoplasts (Czarnecka et al., 2012), but it is not known if the system would behave similarly in stably transformed plants. Another application of synthetic positive feedback circuit is stimulus memory, in which a response to a brief exposure is maintained well beyond the time the stimulus is present.

## Conclusion

Synthetic biology has moved beyond single-cell systems such as *E. coli* and yeast and is moving into complex multicellular eukaryotic systems, including plants. The field is distinguished from traditional genetic engineering by its use of quantitative characterization of individual genetic parts and its use of predictive computational tools for parts selection and circuit design. While plants represent a virtually limitless opportunity for development of novel synthetic circuits and traits, they pose challenges as well. Full automation of design, assembly, and production of predictable circuits is currently possible in microbes (Nielsen et al., 2016). Plants on the other hand are composed of differentiated cells and tissues, each representing potentially unique regulatory or developmental contexts to introduced synthetic genetic circuits. Further, plants have evolved to be highly sensitive to environmental influences such as light or temperature, any of which can impact the quantitative function of individual parts or whole circuits. Mathematical modeling will need to account for the variable contexts a gene might face in a plant, and computerized parts selection will produce circuits with predictable function in a variety of tissues and environments. The ability to collect detailed quantitative data on parts function in cells and tissues is still in its early stages, but is the key to the future of adapting plants to serve human needs in ways that nature never achieved. Beyond the design and assembly of new plant circuits is the practical issue of physically assembling the often very large DNA fragments. Fortunately, the ability to synthesize very large DNA fragments has advanced rapidly (Annaluru et al., 2014; Gibson and Venter, 2014), placing circuits of size and complexity not previously thought possible within our grasp.

## Author Contributions

DM wrote the majority of the text, with valuable input from JM. All authors contributed to the article and approved the submitted version.

### Conflict of Interest

JM is co-inventor on a patent application using plant genetic parts.

The remaining author declares that the research was conducted in the absence of any commercial or financial relationships that could be construed as a potential conflict of interest.
